# Cognitive Telerehabilitation for Older Adults With Neurodegenerative Diseases in the COVID-19 Era: A Perspective Study

**DOI:** 10.3389/fneur.2020.623933

**Published:** 2021-01-14

**Authors:** Sara Bernini, Fabrizio Stasolla, Silvia Panzarasa, Silvana Quaglini, Elena Sinforiani, Giorgio Sandrini, Tomaso Vecchi, Cristina Tassorelli, Sara Bottiroli

**Affiliations:** ^1^Scientific Institute for Research, Hospitalization and Healthcare (IRCCS) Mondino Foundation, Pavia, Italy; ^2^Faculty of Law, Giustino Fortunato University, Benevento, Italy; ^3^Department of Electrical, Computer, and Biomedical Engineering, University of Pavia, Pavia, Italy; ^4^Department of Brain and Behavioral Sciences, University of Pavia, Pavia, Italy

**Keywords:** telerehabilitation, telemedicine, information and communication technologies, COVID-19 pandemic, cognitive impairment, cognitive rehabilitation, cognitive training, neurodegenerative diseases

## Abstract

The COVID-19 pandemic is a global health problem that is radically transforming public and private healthcare organizations around the world, negatively affecting the rehabilitative treatments of non-COVID pathologies as well. In this situation, it becomes crucial to be able to guarantee the continuity of care also to all those patients with neurodegenerative diseases unable to reach healthcare services. Remote communication technologies are gaining momentum as potentially effective options to support health care interventions—including cognitive rehabilitation—while patients can stay safely at home. In this context, we are implementing HomeCoRe (i.e., Home Cognitive Rehabilitation software) in order to offer an innovative approach and a valid support for home-based cognitive rehabilitation in neurodegenerative diseases, such as mild cognitive impairment and early dementia. HomeCoRe has been developed within a research project between engineers and clinicians in order to obtain a usable and safe cognitive rehabilitation tool. This software has multiple advantages for patients and therapists over traditional approaches, as shown in its use in hospital settings. HomeCoRe could then represent an opportunity for accessing cognitive rehabilitation in all those situations where patients and therapists are not in the same location due to particular restrictions, such as COVID-19 pandemic.

## Introduction

With the rise in life expectancy during the last decades, we are witnessing a steady increase in the number of older adults in the total population with a high risk of developing neurodegenerative diseases (1). In particular, among these, dementia represents one of the major health problems in older adults, with progressive deterioration of cognition, daily functioning, and behavior that together lead to disability. This is further exacerbated by the existing link among cognitive decline, hospitalization, and mortality, resulting in a considerable challenge to patients, caregivers, and the health system in term of resources allocation (2). The transitional phase between normal and pathological cognitive aging is a clinical condition called Mild Cognitive Impairment (MCI), which represents a risk factor for the development of dementia (3). Although not all MCI patients progress to dementia, interventions at this pre-dementia stage may be able to reduce/slow down the deterioration along the continuum of MCI and dementia (4).

Because of the limited effectiveness of pharmacological therapies, attempts have been made to provide identification of other factors in patients' care that may delay the onset and slow progression of cognitive decline in MCI. In particular, non-pharmacological interventions have received increasing attention in recent years (5). Particularly, there is evidence that cognitive training is an effective intervention strategy in improving or at least maintaining cognitive level in MCI patients, thus slowing the progression to dementia (6, 7). Cognitive training and enhancement activities can indeed activate brain compensation mechanisms to tackle the physiological and pathological neuro-degeneration processes (8). Traditional cognitive training includes paper-and-pencil exercises usually administered in hospital settings and, less frequently, at patients' homes (9). Since this kind of intervention has some limitations—e.g., time, costs, and patients' accessibility, to name a few—their provision outside the clinical setting is often reduced (10, 11). In recent years, the development of Information and Communication Technologies (ICT) has kindled interest in alternative rehabilitative approaches. In particular, computer-based cognitive training allows one to overcome the limits of traditional paper-and-pencil techniques providing patient-tailored interventions that can be easily delivered not only in-person but also remotely at patients' homes (12). It means that they could simplify the therapist's work in terms of the planning, design, and management of the cognitive intervention also outside from the clinical setting.

To date, unprecedented new challenges to patients' care have been determined by the COVID-19 pandemic, including difficulties accessing routine treatments, such as cognitive rehabilitation, for individuals with neurodegenerative diseases. Hence, in parallel to the increase in the number of studies that claim for ICTs implementation in patient management, their effective integration in the routine clinical practice is still limited (13). The aim of this perspective article is to explore current evidence-based recommendations on the efficacy of ICT-based cognitive rehabilitation to achieve/continue adequate cognitive stimulation in the current pandemic. In this context, it is also offered a perspective about an innovative approach and a valid support for home-based cognitive rehabilitation in neurodegenerative diseases, which is HomeCoRe (i.e., Home Cognitive Rehabilitation software).

## Telemedicine and Telerehabilitation

Telemedicine is defined as an interface in a virtual patient-clinician relationship to provide primary and secondary care by ICT (14). It is not intended to replace the healthcare model based on face-to-face interaction, but rather it is its declination varying according to patients' needs and characteristics (15). Telemedicine could be useful in the management of chronic diseases having high social impact and issues related to continuous long-term care, including diseases related to aging, such as dementia and other neurodegenerative disorders (14).

In particular, telerehabilitation (TR) is a young telemedicine subfield that could be defined as the set of instruments and protocols aimed at providing rehabilitation at a distance (16). Allowing remote delivery of different rehabilitation services in different medical conditions, TR provides benefits for the healthcare system and patients in terms of cost-effectiveness and feasibility for large-scale implementations. TR can use different types of technologies, such as sensor-based technology, tele/video-conference, specific *ad hoc* development software, or virtual reality (17).

Narrowing down the field to neurology, the main pathology treated by TR is stroke followed by traumatic brain injury, multiple sclerosis, and Parkinson's disease (18). For instance, some evidence suggests that physical and speech therapy delivered by TR to post-stroke patients is no worse than conventional in-person interventions in terms of reliability and effectiveness (19, 20). Even if motor and speech/voice impairments have been the main targets of TR (18), the interest for the treatment of other disabilities, such as the cognitive deficit following acquired neurological or neurodegenerative diseases, is growing steadily (21). In this field, the cognitive TR literature is more recent and mostly focuses on treating cognitive impairment in patients with stroke (22), multiple sclerosis (23), and brain tumors (24, 25).

## Cognitive Telerehabilitation in Neurodegenerative Diseases

So far, few studies have been conducted to assess feasibility and efficacy of cognitive TR in older people with neurodegenerative diseases, such as MCI, Alzheimer's disease, and frontotemporal dementia. With the growing interest in this field, many study protocols have recently proliferated in the literature [e.g., (26)]. Only two systematic reviews (27, 28) are available on this topic. Cognitive TR has comparable effects in terms of efficacy, validity, and reliability to conventional in-person rehabilitation. However, as reported by Maresca and colleagues (28), most studies are characterized by small samples and lack of standardized procedures, aims, and targets. Accordingly, further randomized controlled trials (RCT) are strongly needed to improve our knowledge of how to use home-based cognitive TR effectively to delay the progression of cognitive impairment in people with MCI and dementia.

This necessity is further supported by the fact that some concerns have slowed the integration of cognitive TR into clinical practice (29, 30), but the existing literature gives some recommendations to overcome them.

First, the loss of human contact with the clinician and the limited flexibility in the adoption of devices most appropriate for patients' differing needs could hinder adherence to TR (17). Similarly, people with advanced age or cognitive deficit might have poor computer skills and difficulties managing technological devices on their own (31). Furthermore, patients' characteristics, such as hearing and vision impairments and the level and type of cognitive impairment may influence the number of post-rehabilitation benefits. All these factors could in fact be an important cause of distraction, especially for older people who may have little or no experience or confidence using technology (32). Hence, platforms should be developed in order to be accessible and user-friendly; duration and frequency of rehabilitation activities should be tailored according to patients' characteristics (33); therapists should monitor adherence and performance of each session remotely during the whole period of treatment (34). In any case, there is evidence that cognitive TR is a valuable and well-accepted methodology, and comparable effects have been found between TR and in-hospital treatment in terms of global cognitive performance in patients with early phases of cognitive deterioration (35).

Second, even if caregivers are supportive and facilitate adherence to TR in daily routines (36), it is important to avoid their excessive involvement to limit the burden of the approach and to prevent thwarting the benefits of the treatment itself. Furthermore, patients without a compliant caregiver could be excluded by the use of TR, representing a selection bias for this kind of intervention (37). However, there is evidence also about the possibility of using telemedicine devices in MCI patients living alone. In particular, it seems that in this case patients' compliance depends on the level of monitoring he/she remotely receives (38). In addition, it is important to consider that easy-access TR tools can produce benefits (e.g., autonomy, mood, self-efficacy, quality of life, etc.) in patients, with consequent positive effects also for caregivers (39).

With these considerations in mind, TR constitutes a unique opportunity in the field of cognitive rehabilitation. It indeed represents a replacement for in-person treatment or its continuation, providing equitable access to care for patients with neurodegenerative diseases (40, 41). Such an opportunity could be useful not only for older patients with dementia or physical disabilities, but also for those presenting pre-dementia symptoms while of working age or geographically remote. More generally, TR could have a pivotal role in the clinical practice in all those situations where patients and therapists cannot be in the same location, due to patients' requirements or, as in the case of the COVID-19 pandemic, because of particular emergencies.

## Cognitive Telerehabilitation During Covid-19 Pandemic

The COVID-19 pandemic, caused by the SARS-CoV-2 coronavirus, is a global health problem that has radically transformed public and private healthcare organizations around the world (42). This enduring health emergency, and the consequent adaptation of healthcare facilities, negatively influences the rehabilitative treatments of non-COVID pathologies, with an impact on the quality of life of patients (especially those with cognitive symptoms) and their families. In particular, social isolation, a long confinement period, and personal experiences combined with pre-existing diseases may play an important role in exacerbating cognitive decline and dementia (43). As an urgent response to provide continuity of care and social connectedness during the COVID-19 pandemic, new alternative options of cognitive rehabilitation are needed. To this end, remote communication technologies are increasingly considered as potentially effective options to support healthcare interventions, among which is cognitive rehabilitation, directly at the patient's home, reducing risks of possible infections (44–46). Aging *per se* is, in fact, associated with vulnerabilities of a physical, psychosocial, and environmental nature (47), determining more comorbidities and hospitalizations and, as a consequence, an increasing chance of being infected with COVID-19 (48). Such a susceptibility to morbidity and mortality from COVID-19 becomes more pronounced in those older adults with dementia (49). Hence, rehabilitation via remote communication technologies may represent a viable alternative tool to access care while reducing the risk of COVID-19 infection and avoiding unnecessary travel and discomfort to the patient and other family members (50).

Within this framework, cognitive TR may be viewed as a valid recovery tool (51) deriving from the reshape of cognitive rehabilitation with the use of technologies (52). Hence, based on these promising results and forced by the COVID-19 pandemic contingency, new studies and a larger diffusion of cognitive TR approaches are expected (53–55). To date, most efforts have been devoted to using telemedicine/telerehabilitation to address patients' recovery after COVID-19, which is very important in order to monitor and manage resulting deficits (56–61). For instance, Salawu and colleagues (60) have proposed a multidisciplinary TR program for patients with COVID-19 discharged from hospitals with residual rehabilitation needs. However, telemedicine and telerehabilitation should be implemented also in non-COVID patients in various settings of neurological care (36, 62, 63). From this perspective, Motolese and colleagues (36) explored the feasibility of a smartphone application for monitoring motor and cognitive performance of non-demented Parkinson's disease outpatients during the lockdown. Ramalho and colleagues (64) proposed a protocol of telemental healthcare to be applied to populations with different levels of needs, including older adults in need of constant home-based assistance. Again, in the field of pathological aging, other recommendations pertain to the management of behavioral and psychological symptoms of dementia or long-term care of older adults living in nursing homes via telemedicine (65). To the best of our knowledge, no experience has been published on the use of TR in older adults with cognitive impairment during COVID-19, even if strongly recommended (53–55).

## A Perspective for Future Cognitive Rehabilitation: HomeCoRe

During the past years, we have implemented and used a computer-supported cognitive training program (Cognitive Rehabilitation—CoRe—software) for in-person sessions in the hospital setting (66, 67). CoRe has been developed within a research project between engineers and clinicians. We reported that CoRe was safe and effective with respect to cognition in inpatients with Parkinson Disease-Mild Cognitive Impairment (68, 69) and also in older adults with other forms of early cognitive impairment (70). Following these encouraging results observed in the hospital setting, we recently developed a TR version of CoRe that allows the provision of treatment at patients' homes: HomeCoRe (71).

**Figure 1:**
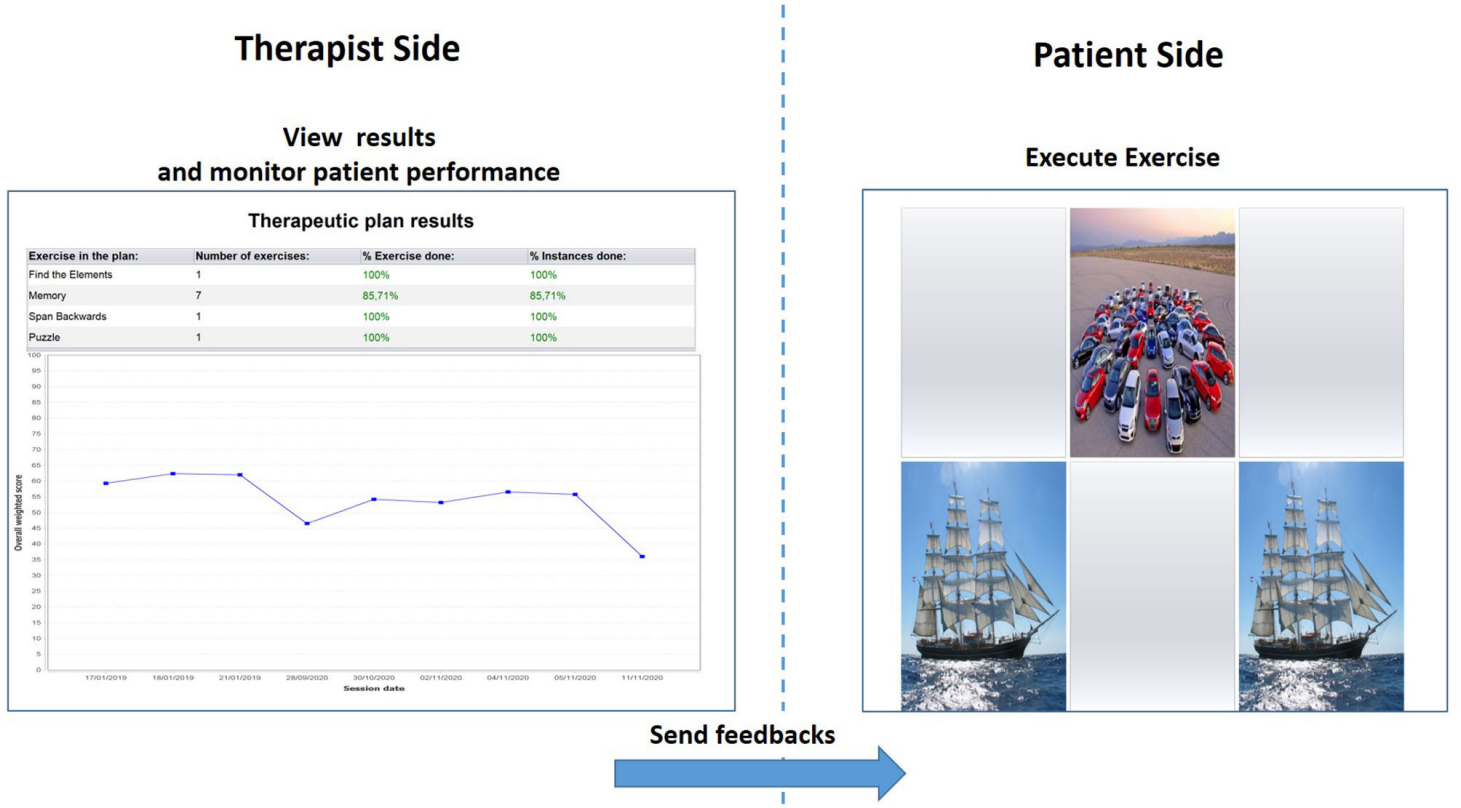
Therapist interface for monitoring results and patient performance in terms of overall Weighted Score **(left)** and patient interface for the execution of the memory exercise **(right)**.

HomeCoRe is a patient-tailored intervention aimed at stimulating several cognitive abilities (e.g., logical-executive functions, attention/processing speed, working memory, and episodic memory) through a series of sessions of 2D exercises planned remotely for multiple advantages for therapists and patients. It is timesaving, ready to use, and able to set exercises for each training session automatically. Exercises take place in an adaptive mode. In particular, during their dynamic generation, the individual patients' performance data (accuracy and number of aids required) are analyzed in order to set the appropriate difficulty level. Furthermore, for each exercise and each level, thresholds are defined so as to allow difficulty levels to be progressively increased. In addition, the system calculates an “overall weighted score (WS),” taking into account the correctness of the answers, the execution time, and the difficulty of the exercises. The WS informs the therapist about each patient's performance in a single value. Hence, WS represents a useful and advantageous index that can be used to assess and monitor both the overall outcome of a training session and the global trend of the rehabilitation (Figure 1).

**Figure 2:**
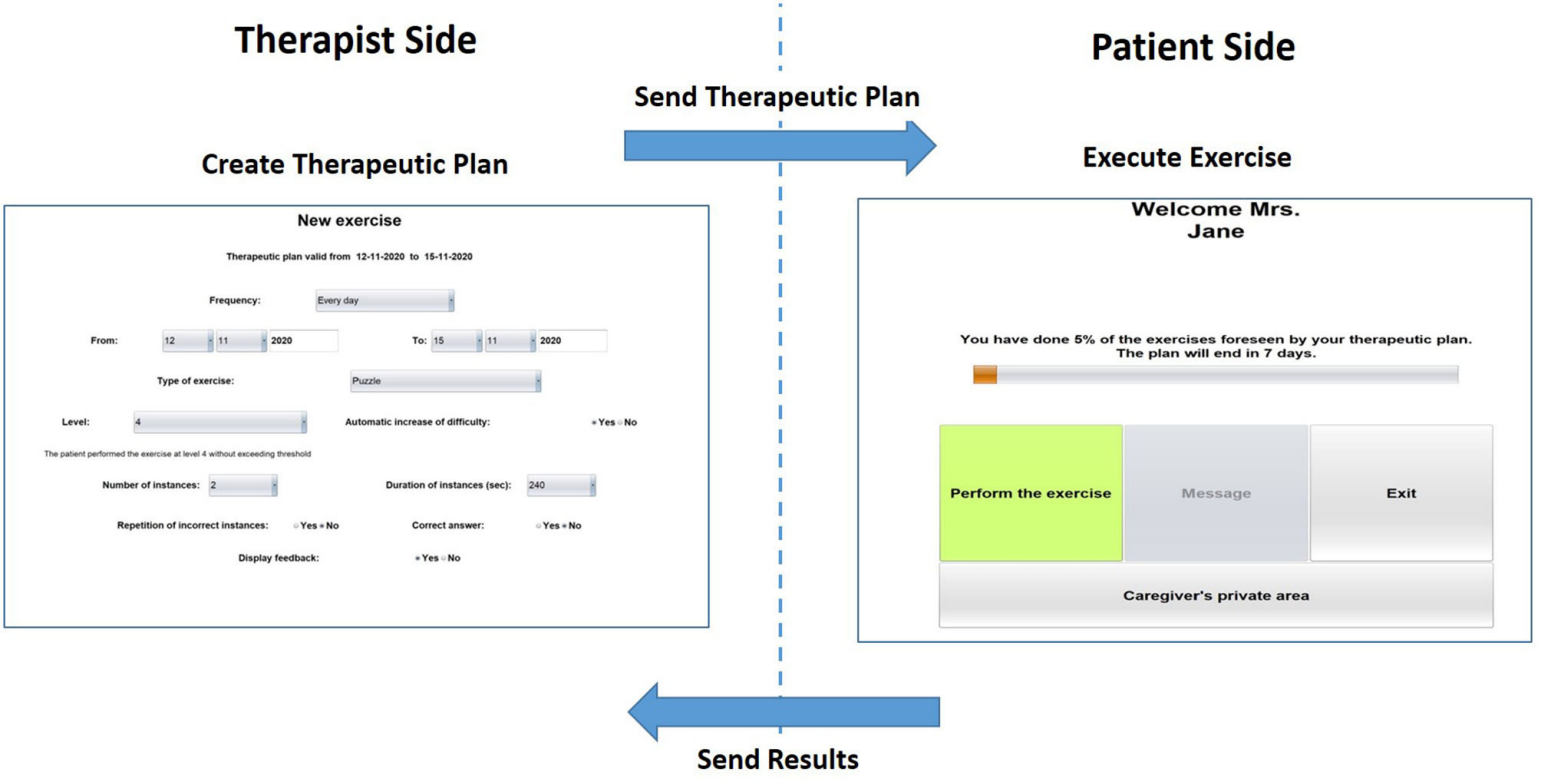
Home page of the therapist-side of the interface for setting the requirements for the exercise plan **(left)** and home page of the patient/caregiver-side of the interface **(right)**.

The HomeCoRe architecture includes two main components, the therapist side and the patient/caregiver side, as well as the communication channels between them. The therapist side of the interface allows the remote setting and monitoring of all requirements of the treatment plan (e.g., frequency and duration of the plan, types of exercise, and difficulty level) (see Figure 2). The patients/caregiver side of the interface is very simple to use, and it allows for viewing and executing the exercises of the day and communicating with the therapist (see Figure 2).

In a recent work (71), we interviewed and surveyed inpatients to investigate their willingness to continue rehabilitation at home by using HomeCoRe after discharge. Caregivers were also interviewed, due to their role in both supporting and motivating patients. The survey results showed that most of both patient participants and caregivers appreciated HomeCoRe and intended to have a further home commitment. Subsequently, we tested the functionality and usability of HomeCoRe by using in-hospital workstations that simulated home sessions. Currently, we are carrying out a pilot study on a small sample of patients in the early stage of cognitive deterioration testing HomeCoRe directly at patients' homes. This will allow evaluating both patients' and caregivers' experience (e.g., compliance, benefits) and the cognitive effects of HomeCoRe rehabilitation. The output of this pilot study will inform a randomized clinical trial to explore the cost-effectiveness of cognitive telerehabilitation via HomeCoRe compared with in-person cognitive rehabilitation in patients with neurodegenerative diseases.

HomeCoRe promises to qualify as an innovative approach and a valid support for cognitive rehabilitation in neurodegenerative diseases. In addition, as a TR tool, HomeCoRe will allow extending the duration of the rehabilitation treatment of inpatients beyond the hospital discharge, which often coincides with treatment interruption, due to the scarcity of healthcare personnel for homecare. It also offers a unique opportunity to deliver cognitive rehabilitation to people who live in remote areas or who cannot reach healthcare services due to physical impairments or particular restrictions, such as the COVID-19 pandemic.

## Conclusion

Due to the progressive aging of our population, the number of people with MCI or dementia is expected to grow consistently, with a social impact and economic burden on the healthcare system. Therefore, the World Health Organization (WHO) stresses taking global action against cognitive decline and dementia, encouraging governments worldwide to focus on prevention and to improve healthcare services. In addition, in line with the new health and social order that has been determined since the COVID-19 pandemic, it is crucial to offer a cognitive rehabilitation modality that can be used directly at home, in a condition of distance and safety for both family members and the patient her/himself. To this end, remote communication technologies are increasingly regarded as potentially effective options—with the appropriate recommendations (29, 30)—to support cognitive rehabilitation (53–55). In this framework, HomeCoRe is software for cognitive rehabilitation in neurodegenerative diseases that could be incorporated into clinical routine protocols as a complementary non-pharmacological therapy to support the continuum of care from the hospital to the patient's home.

In conclusion, the COVID-19 pandemic has determined new chances to embrace technology allowing people to maintain their connection with the outside world during isolation (72, 73). Such opportunities can also be extended to the delivery of care for neurodegenerative diseases, producing a technological evolution in the healthcare system (74–76) and dementia practice (53–55, 77), in the coming years.

## Data Availability Statement

The original contributions generated in the study are included in the article/supplementary material, further inquiries can be directed to the corresponding author.

## Ethics Statement

The studies involving human participants were reviewed and approved by San Matteo Ethics Committee. The patients/participants provided their written informed consent to participate in this study.

## Author Contributions

SBe and SBo have conceived the work. All authors listed have made a substantial, direct and intellectual contribution to the work and approved it for publication.

## Conflict of Interest

The authors declare that the research was conducted in the absence of any commercial or financial relationships that could be construed as a potential conflict of interest.
